# Comparison of clinical characteristics and prognosis in endometrial carcinoma with different pathological types: a retrospective population-based study

**DOI:** 10.1186/s12957-023-03241-0

**Published:** 2023-11-21

**Authors:** Gong Zhang, Fangfang Nie, Weinan Zhao, Pin Han, Jing Wen, Xiaoran Cheng, Weijia Wu, Qianwen Liu, Yi Sun, Yuanpei Wang, Yuchen Liu, Fang Ren

**Affiliations:** 1 Department of Hepatobiliary and Pancreatic Surgery, The First Affiliated Hospital of Zhengzhou University, Zhengzhou, Henan China; 2 Department of Obstetrics and Gynecology, The First Affiliated Hospital of Zhengzhou University, Zhengzhou, Henan China; 3 Department of Obstetrics and Gynecology, Xuchang Central Hospital, Xuchang, Henan China; 4 Department of Obstetrics and Gynecology, Luoyang Maternal and Child Health Care Hospital, Luoyang, Henan China

**Keywords:** Endometrial carcinoma, Uterine endometrioid carcinoma, Uterine serous carcinoma, Uterine mixed carcinoma, Uterine clear cell carcinoma, Prognosis

## Abstract

**Background:**

Endometrial carcinoma (EC) is the second most common gynecological malignancy, and the differences between different pathological types are not entirely clear. Here, we retrospectively collected eligible EC patients to explore their differences regarding clinical characteristics and prognosis.

**Methods:**

Five hundred seventy EC patients from the First Affiliated Hospital of Zhengzhou University were included. Prognostic factors were measured using the univariate/multivariate Cox models. Overall survival (OS) and progression-free survival (PFS) were the primary and secondary endpoints, respectively.

**Results:**

In total, 396 patients with uterine endometrioid carcinoma (UEC), 106 patients with uterine serous carcinoma (USC), 34 patients with uterine mixed carcinoma (UMC), and 34 patients with uterine clear cell carcinoma (UCCC) were included. Comparison of baseline characteristics revealed patients diagnosed with UEC were younger, had more early clinical stage, and had lower incidence of menopause and lymph node metastasis. Compared to UEC, other pathological EC obtained more unfavorable OS (UCCC: HR = 12.944, 95%CI = 4.231–39.599, *P* < 0.001; USC: HR = 5.958, 95%CI = 2.404–14.765, *P* < 0.001; UMC: HR = 1.777, 95%CI = 0.209–15.114, *P* = 0.599) and PFS (UCCC: HR = 8.696, 95%CI = 1.972–38.354, *P* = 0.004; USC: HR = 4.131, 95%CI = 1.243–13.729, *P* = 0.021; UMC: HR = 5.356, 95%CI = 0.935–30.692, *P* = 0.060). Compared with UEC patients, the OS of UCCC patients in stage I–II and USC patients in stage III–IV were significantly worse, while UMC patients in stage I–II favored poorer PFS. The OS of UCCC patients receiving no postoperative adjuvant therapy or chemotherapy alone were significantly worse.

**Conclusions:**

The baseline characteristics of UEC and other rare EC types varied greatly, and the prognostic significance of different pathological types on EC patients depended on clinical tumor stages and therapeutic options.

**Supplementary Information:**

The online version contains supplementary material available at 10.1186/s12957-023-03241-0.

## Background

EC is still one of the most fatal malignant tumors, resulting in 417,367 new cases and 97,370 fatalities in 2020 worldwide [1]. Indeed, the etiological factors of EC remain uncharted. It is generally believed that EC can be divided into two different types based on pathogenesis and biological behavior characteristics, namely estrogen-dependent (type I) and estrogen-independent (type II). Among these, type I is predominately UEC, accounting for 80% of EC cases. While type II is mainly composed of different pathological types (e.g., USC, UCCC, UMC), accounting for 15–20% of all EC cases [2–4]. Patients with type II EC obtain a lower 5-year survival rate compared to those with type I EC, and it is estimated that type II EC causes over 45% of EC-related deaths [5–8]. However, there are few studies comparing the differences in baseline characteristics and prognosis between rare pathological subtypes and type I EC simultaneously, which needs further exploration.

Previous studies have revealed that type I and type II EC displayed completely different genomic and molecular characteristics, which may affect a patient’s prognosis by reshaping biological behavior and drug response [9, 10]. For example, genomic variations of PTEN, PIK3CA, PIK3R1, KRAS, ARID1A, and CTNNB1 are more common in type I EC, while mutations in TP53, PPP2R1A, PIK3CA, and FBXW7 are more common in type II EC [11, 12]. Previous studies have identified specific risk factors for type I (e.g., estrogen exposure, obesity, nulliparity) and type II (e.g., old age, menopause) EC cohorts [13]. Nevertheless, compared to grade 1/2 UEC, whether different pathological types can be considered as prognostic factors has rarely been investigated in EC cohorts.

Herein, we revealed prognostic factors and prognostic (OS and PFS) differences of EC patients with different pathological types by retrospectively collecting EC samples from the Department of Obstetrics and Gynecology of the First Affiliated Hospital of Zhengzhou University.

## Methods

### Screening of eligible EC patients

This retrospective study was approved by the Ethics Committee of the First Affiliated Hospital of Zhengzhou University (permission number: 2023-KY-0350–002). We retrieved the hospital’s case system and identified those EC cases diagnosed with USC, UCCC, UMC, or grade 1/2 UEC, as potentially eligible patients from 2009 to 2021. The pathologic diagnosis of all included patients was reviewed by two senior pathologists. The following clinical data was collected: diagnosis data, menopausal status, age, height, body weight, pathological type, treatment program (surgery, chemotherapy, radiotherapy, etc.), the status of lymph node metastasis and cervix involvement, the depth of myometrial infiltration, the status of survival and recurrence. Patients with other malignant tumors or missing prognostic information were excluded. The clinical stage of eligible patients was redefined according to the 2009 International Federation of Gynecology and Obstetrics (FIGO) staging system.

### Processing of clinical data

In our study, OS was defined as the primary endpoint, which referred to the time from diagnosis to death or last follow-up. PFS, the secondary endpoint, was defined as the time from diagnosis to the first reported recurrence or last follow-up. Only patients with accurate OS data were included in our analysis. Subgroup analyses were performed based on the patient’s clinical stage and treatment programs.

### Statistics analysis

The comparison of baseline characteristics between UCCC, USC, UMC, and UEC groups was performed using the R stats (version 4.2.1). Kaplan–Meier curves were plotted using the R survival (version 3.3.1), and the Cox regression test was used to conduct survival analyses. Prognostic factors were measured using the univariate/multivariate Cox models. *P* value less than 0.05 was considered statistically significant.

## Results

### Comparison of baseline characteristics between different pathological EC subtypes

According to the mentioned inclusion and exclusion criteria, 570 EC patients (570/2056) were included for subsequent analysis. Among them, 396 grade 1/2 UEC patients, 106 USC patients, 34 UMC patients, and 34 UCCC patients. Further comparison displayed vast differences in demographics and clinical characteristics between different pathological EC subtypes. In general, patients with USC, UCCC, or UMC were all at an older age and had a higher incidence of menopause status than those with UEC (Table 1). Except for UMC, patients with USC and UCCC were diagnosed at the more advanced clinical stage and had a higher incidence of lymph node metastasis (Table 1). Another interesting finding was that only patients with USC had higher rates of myometrial infiltration and cervix involvement compared to patients with UEC (Table 1). Overall, patients with UEC (median: 55.65 months) shared longer survival time compared to USC (median: 36.83 months), UCCC (median: 38.02 months), or UMC (median: 45.87 months), the same was true for PFS (Table 2). The rates of UEC (9.1%) patients receiving radiotherapy were significantly lower than those of UCCC (32.4%), USC (40.6%), and UMC (29.4%) patients, respectively. The rates of UEC (41.7%) patients receiving chemotherapy were significantly lower than those of USC (76.4%), and UMC (79.4%) patients, respectively (Table 2).

**Table 1:** Baseline characteristics of included patients with different pathological types

Characteristics	UEC	UCCC	USC	UMC	*P*	*P*^a^	*P*^b^	*P*^c^
*n*	396	34	106	34				
Age, mean ± sd	54.705 ± 8.7763	64.088 ± 9.0833	61.453 ± 8.5213	58 ± 5.1757	** < 0.001**	** < 0.001**	** < 0.001**	**0.002**
Menopause, *n* (%)					** < 0.001**	** < 0.001**	** < 0.001**	** < 0.001**
Yes	235 (59.3%)	31 (91.2%)	95 (89.6%)	32 (94.1%)				
No	150 (37.9%)	3 (8.8%)	10 (9.4%)	2 (5.9%)				
Unknown	11 (2.8%)	0 (0%)	1 (0.9%)	0 (0%)				
BMI, median (IQR)	25.462 (23.422, 28.134)	26.531 (22.481, 28.125)	24.654 (22.638, 27.447)	25.1 (23.508, 26.667)	0.480	0.933	0.162	0.430
Stage, *n* (%)					** < 0.001**	** < 0.001**	** < 0.001**	< 0.627
IV	2 (0.5%)	1 (2.9%)	11 (10.4%)	0 (0%)				
III	27 (6.8%)	6 (17.6%)	26 (24.5%)	4 (11.8%)				
II	6 (1.5%)	2 (5.9%)	7 (6.6%)	1 (2.9%)				
I	361 (91.2%)	21 (61.8%)	59 (55.7%)	29 (85.3%)				
Unknown	0 (0%)	4 (11.8%)	3 (2.8%)	0 (0%)				
Myometrial infiltration (> = 1/2),* n* (%)					** < 0.001**	0.090	** < 0.001**	0.182
Yes	70 (17.7%)	4 (11.8%)	49 (46.2%)	10 (29.4%)				
No	297 (75%)	24 (70.6%)	50 (47.2%)	23 (67.6%)				
Unknown	29 (7.3%)	6 (17.6%)	7 (6.6%)	1 (2.9%)				
Cervix involvement, *n* (%)					** < 0.001**	0.142	** < 0.001**	0.073
Yes	18 (4.5%)	4 (11.8%)	23 (21.7%)	3 (8.8%)				
No	333 (84.1%)	25 (73.5%)	80 (75.5%)	31 (91.2%)				
Unknown	45 (11.4%)	5 (14.7%)	3 (2.8%)	0 (0%)				
Lymph node metastasis, *n* (%)					** < 0.001**	**0.008**	** < 0.001**	0.053
Yes	19 (4.8%)	6 (17.6%)	31 (29.2%)	2 (5.9%)				
No	299 (75.5%)	23 (67.6%)	62 (58.5%)	31 (91.2%)				
Unknown	78 (19.7%)	5 (14.7%)	13 (12.3%)	1 (2.9%)				

*UEC* uterine endometrioid carcinoma, *USC* uterine serous carcinoma, *UMC* uterine mixed carcinoma, *UCCC* uterine clear cell carcinoma, *BMI* body mass index

^a^*P* UEC versus UCCC

^b^*P* UEC versus USC

^c^*P* UEC versus UMC

**Table 2:** Adjuvant treatment regimens and prognosis of included patients with different pathological types

Characteristics	UEC	UCCC	USC	UMC	*P*	*P*^a^	*P*^b^	*P*^c^
*n*	396	34	106	34				
Chemotherapy, *n* (%)					** < 0.001**	0.052	** < 0.001**	** < 0.001**
Yes	165 (41.7%)	20 (58.8%)	81 (76.4%)	27 (79.4%)				
No	231 (58.3%)	14 (41.2%)	25 (23.6%)	7 (20.6%)				
Radiotherapy,* n* (%)					** < 0.001**	** < 0.001**	** < 0.001**	** < 0.001**
Yes	36 (9.1%)	11 (32.4%)	43 (40.6%)	10 (29.4%)				
No	360 (90.9%)	23 (67.6%)	63 (59.4%)	24 (70.6%)				
OS, *n* (%)					** < 0.001**	** < 0.001**	** < 0.001**	1.000
Alive	384 (97%)	25 (73.5%)	81 (76.4%)	33 (97.1%)				
Dead	12 (3%)	9 (26.5%)	25 (23.6%)	1 (2.9%)				
OS-time(months), median (IQR)	55.65 (46.42, 71.13)	38.02 (27.36, 55.58)	36.83 (25.48, 60.08)	45.87 (34.88, 62.93)	** < 0.001**	** < 0.001**	** < 0.001**	**0.001**
PFS,* n* (%)					** < 0.001**	** < 0.001**	** < 0.001**	0.227
Stable	380 (96%)	24 (70.6%)	75 (70.8%)	31 (91.2%)				
Recurrent	9 (2.3%)	5 (14.7%)	11 (10.4%)	2 (5.9%)				
Unknown	7 (1.8%)	5 (14.7%)	20 (18.9%)	1 (2.9%)				
PFS-time(months), median (IQR)	55.90 (46.43, 71.13)	41.10 (31.33, 56.53)	40.93 (26.53, 62.53)	45.67 (36.83, 62.97)	** < 0.001**	** < 0.001**	** < 0.001**	** < 0.001**

*UEC* uterine endometrioid carcinoma, *USC* uterine serous carcinoma, *UMC* uterine mixed carcinoma, *UCCC* uterine clear cell carcinoma, *OS* overall survival, *PFS* progression-free survival

^a^*P* UEC versus UCCC

^b^*P* UEC versus USC

^c^*P* UEC versus UMC

### Comparison of prognosis between different pathological EC subtypes

**Fig. 1:**
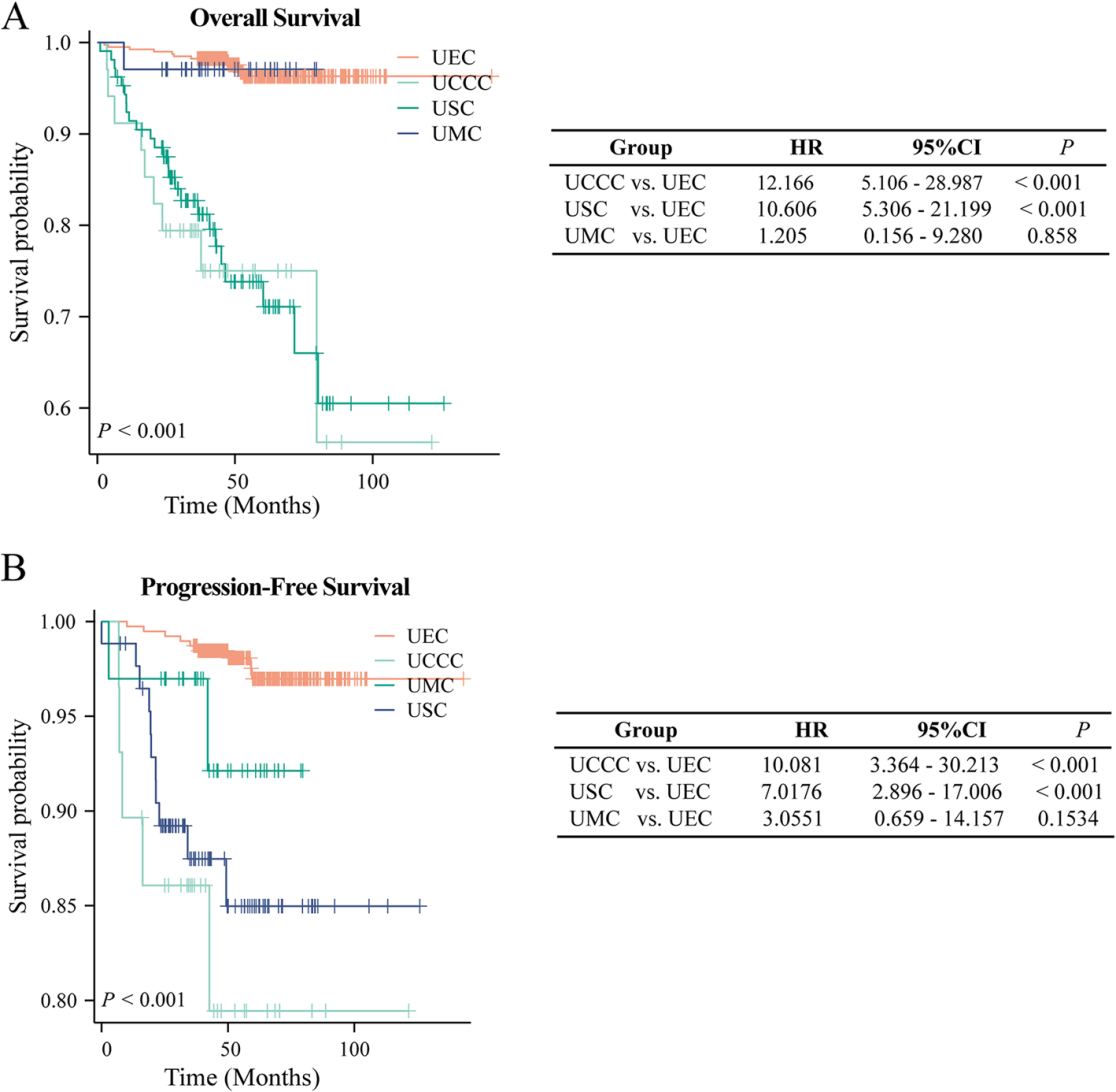
Kaplan–Meier curves of included EC patients with different pathological types. **A** Prognostic significance of different pathological types on overall survival. **B** Prognostic significance of different pathological types on progression-free survival

To measure the prognostic effects of different pathological types on prognosis in EC (PFS and OS), we plotted Kaplan–Meier survival curves based on collected data. As shown in Fig. 1, patients with USC or UCCC significantly favored poorer OS and PFS compared to those with UEC. Further univariate and multivariate logistic regression analyses were used to identify independent factors affecting patients’ prognosis in the entire EC population included. For OS, age (HR = 1.050, 95%CI = 1.010–1.091, *P* = 0.014) and myometrial infiltration (> = 1/2) (HR = 3.390, 95%CI = 1.506–7.631, *P* = 0.003) were independent factors associated with patients’ unfavorable prognosis in EC (Table 3). Except for UMC (HR = 1.777, 95%CI = 0.209–15.114, *P* = 0.599), patients with USC (HR = 5.958, 95%CI = 2.404–14.765, *P* < 0.001), and UCCC (HR = 12.944, 95%CI = 4.231–39.599, *P* < 0.001) favored unfavorable OS (Table 3).

**Table 3:** Univariate and multivariate Cox regression analysis for OS

Characteristics	No	Univariate analysis		Multivariate analysis	
		Hazard ratio (95% CI)	*P*	Hazard ratio (95% CI)	*P*
Age	570	1.108 (1.075—1.142)	** < 0.001**	1.050 (1.010—1.091)	**0.014**
Menopause	570		** < 0.001**		
No	165	Reference		Reference	
Yes	393	10.403 (2.523–42.893)	**0.001**	2.758 (0.580–13.128)	0.202
Unknown	12	0.000 (0.000–Inf)	0.996	0.000 (0.000–Inf)	0.997
BMI	255	1.025 (0.927–1.134)	0.627		
Chemotherapy	570		0.857		
No	277	Reference			
Yes	293	0.949 (0.535–1.682)	0.857		
Radiotherapy	570		0.448		
No	470	Reference			
Yes	100	1.320 (0.656–2.655)	0.436		
Stage	570		** < 0.001**		
I	470	Reference		Reference	
II	16	2.926 (0.684–12.522)	0.148	1.177 (0.223–6.217)	0.848
III	63	6.122 (3.088–12.137)	** < 0.001**	1.075 (0.218–5.300)	0.929
IV	14	28.979 (12.307–68.238)	** < 0.001**	3.873 (0.936–16.032)	0.062
Unknown	7	17.109 (5.030–58.193)	** < 0.001**	0.489 (0.062–3.839)	0.496
Myometrial infiltration (> = 1/2)	570		** < 0.001**		
No	394	Reference		Reference	
Yes	133	6.269 (3.300–11.910)	** < 0.001**	3.390 (1.506–7.631)	**0.003**
Unknown	43	3.764 (1.354–10.463)	**0.011**	0.791 (0.138–4.539)	0.793
Cervix involvement	570		** < 0.001**		
No	469	Reference		Reference	
Yes	48	4.992 (2.526–9.866)	** < 0.001**	0.912 (0.390–2.133)	0.831
Unknown	53	2.850 (1.293–6.283)	**0.009**	4.820 (1.300–17.866)	**0.019**
Lymph node metastasis	570		** < 0.001**		
No	415	Reference		Reference	
Yes	58	10.920 (5.600–21.294)	** < 0.001**	3.333 (0.761–14.604)	0.110
Unknown	97	3.401 (1.609–7.191)	**0.001**	4.153 (1.623–10.626)	**0.003**
Pathological type	570		** < 0.001**		
UEC	396	Reference		Reference	
UCCC	34	12.166 (5.106–28.987)	** < 0.001**	12.944 (4.231–39.599)	** < 0.001**
USC	106	10.606 (5.306–21.199)	** < 0.001**	5.958 (2.404–14.765)	** < 0.001**
UMC	34	1.205 (0.156–9.280)	0.858	1.777 (0.209–15.114)	0.599

*UEC* uterine endometrioid carcinoma, *USC* uterine serous carcinoma, *UMC* uterine mixed carcinoma, *UCCC* uterine clear cell carcinoma, *BMI* body mass index, *OS* overall survival

For PFS, age (HR = 1.091, 95%CI = 1.021–1.166, *P* = 0.010), myometrial infiltration (> = 1/2) (HR = 3.788, 95%CI = 1.255–11.427, *P* = 0.018), and cervix involvement (HR = 6.253, 95%CI = 1.620–24.138, *P* = 0.008) had negative effects on patient prognosis. Meanwhile, patients with USC (HR = 4.131, 95%CI = 1.243–13.729, *P* = 0.021) and UCCC (HR = 8.696, 95%CI = 1.972–38.354, *P* = 0.004) still favored unfavorable PFS (Table 4).

**Table 4:** Univariate and multivariate Cox regression analysis for PFS

Characteristics	No	Univariate analysis		Multivariate analysis	
		Hazard ratio (95% CI)	*P*	Hazard ratio (95% CI)	*P*
Age	536	1.097 (1.054–1.143)	** < 0.001**	1.091 (1.021–1.166)	**0.010**
Menopause	536		**0.027**		
No	162	Reference		Reference	
Yes	363	3.610 (1.084–12.025)	**0.037**	0.539 (0.109–2.677)	0.450
Unknown	11	0.000 (0.000–Inf)	0.997	0.000 (0.000–Inf)	0.998
BMI	237	0.962 (0.846–1.095)	0.558		
Chemotherapy	536		0.066		
No	261	Reference		Reference	
Yes	275	2.125 (0.923–4.892)	0.076	0.609 (0.207–1.791)	0.368
Radiotherapy	536		**0.002**		
No	440	Reference		Reference	
Yes	96	3.684 (1.692–8.024)	**0.001**	1.096 (0.375–3.209)	0.867
Stage	536		**0.025**		
I	457	Reference		Reference	
II	15	3.744 (0.861–16.290)	0.078	0.524 (0.073–3.788)	0.522
III	53	4.193 (1.724–10.197)	**0.002**	0.000 (0.000–Inf)	0.997
IV	7	5.798 (0.759–44.274)	0.090	0.000 (0.000–Inf)	0.998
Unknown	4	0.000 (0.000–Inf)	0.997	0.143 (0.000–Inf)	1.000
Myometrial infiltration (> = 1/2)	536		** < 0.001**		
No	384	Reference		Reference	
Yes	114	5.562 (2.523–12.262)	** < 0.001**	3.788 (1.255–11.427)	**0.018**
Unknown	38	0.000 (0.000–Inf)	0.997	0.000 (0.000–Inf)	0.998
Cervix involvement	536		** < 0.001**		
No	448	Reference		Reference	
Yes	41	9.638 (4.224–21.994)	** < 0.001**	6.253 (1.620–24.138)	**0.008**
Unknown	47	2.232 (0.635–7.839)	0.210	4.724 (0.929–24.014)	0.061
Lymph node metastasis	536		**0.001**		
No	406	Reference		Reference	
Yes	44	5.487 (2.344–12.847)	** < 0.001**	406749342.7904 (0.000–Inf)	0.997
Unknown	86	0.598 (0.138–2.602)	0.493	1.044 (0.204–5.340)	0.959
Pathological type	536		** < 0.001**		
UEC	389	Reference		Reference	
UCCC	29	10.192 (3.400–30.548)	** < 0.001**	8.696 (1.972–38.354)	**0.004**
USC	85	6.432 (2.603–15.895)	** < 0.001**	4.131 (1.243–13.729)	**0.021**
UMC	33	3.070 (0.663–14.228)	0.152	5.356 (0.935–30.692)	0.060

*UEC* uterine endometrioid carcinoma, *USC* uterine serous carcinoma, *UMC* uterine mixed carcinoma, *UCCC* uterine clear cell carcinoma, *BMI* body mass index, *PFS* progression-free survival

### Subgroup analysis based on patients’ clinical stages and postoperative adjuvant therapy

To measure whether the effects of identified prognostic factors for EC patients change in different clinical stages or treatments group, we further divided all patients into two subtypes according to their clinical stages and postoperative adjuvant therapy. For EC patients in clinical stage I–II, age (HR = 1.142, 95%CI = 1.078–1.210, *P* < 0.001) and myometrial infiltration (> = 1/2) (HR = 3.316, 95%CI = 1.075–10.230, *P* = 0.037) were independent prognostic factors for OS, and only patients with UCCC (HR = 4.799, 95%CI = 1.121–20.546, *P* = 0.035) favored poorer prognosis compared to those with UEC (Table 5). For EC patients in clinical stage III-IV, radiotherapy (HR = 0.144, 95%CI = 0.044–0.464, *P* = 0.001) and lymph node metastasis (HR = 10.666, 95%CI = 1.303–87.304, *P* = 0.027) had different effects on OS. Patients with USC (HR = 5.950, 95%CI = 1.613–21.951, *P* = 0.007) achieved worse OS compared to those with UEC (Table 6). Interestingly, only patients with UMC (HR = 6.896, 95%CI = 1.078–44.122, *P* = 0.041) in stage I–II favored poorer PFS compared to those with UEC (Supplementary Table S1 and S2).

**Table 5:** Univariate and multivariate Cox regression for OS of stage I–II

Characteristics	No	Univariate analysis		Multivariate analysis	
		Hazard ratio (95% CI)	*P*	Hazard ratio (95% CI)	*P*
Age	486	1.150 (1.095–1.207)	** < 0.001**	1.142 (1.078–1.210)	** < 0.001**
BMI	197	1.117 (0.974–1.280)	0.114		
Chemotherapy	486		0.401		
No	257	Reference			
Yes	229	0.696 (0.297—1.634)	0.405		
Radiotherapy	486		0.071		
No	422	Reference		Reference	
Yes	64	2.551 (0.998–6.520)	0.050	1.382 (0.478–3.998)	0.550
Myometrial infiltration (> = 1/2)	486		**0.003**		
No	369	Reference		Reference	
Yes	83	4.721 (2.003–11.131)	** < 0.001**	3.316 (1.075–10.230)	**0.037**
Unknown	34	1.193 (0.152–9.342)	0.866	0.857 (0.089–8.284)	0.894
Cervix involvement	486		0.061		
No	421	Reference		Reference	
Yes	22	3.994 (1.155–13.810)	**0.029**	1.657 (0.392–7.011)	0.493
Unknown	43	2.773 (0.917–8.391)	0.071	4.522 (0.961–21.270)	0.056
Lymph node metastasis	486		0.106		
Unknown	86	Reference			
No	400	0.457 (0.186–1.121)	0.087		
Pathological type	486		** < 0.001**		
UEC	367	Reference		Reference	
UCCC	23	9.390 (2.878–30.641)	** < 0.001**	4.799 (1.121–20.546)	**0.035**
USC	66	6.059 (2.332–15.745)	** < 0.001**	2.996 (0.852–10.529)	0.087
UMC	30	1.719 (0.217–13.618)	0.608	2.493 (0.278–22.353)	0.414

*UEC* uterine endometrioid carcinoma *USC* uterine serous carcinoma, *UMC* uterine mixed carcinoma, *UCCC* uterine clear cell carcinoma, *BMI* body mass index, *OS* overall survival

**Table 6:** Univariate and multivariate Cox regression for OS of stage III–IV

Characteristics	No	Univariate analysis		Multivariate analysis	
		Hazard ratio (95% CI)	*P*	Hazard ratio (95% CI)	*P*
Age	77	1.042 (1.000—1.085)	0.052	0.977 (0.915–1.043)	0.480
Menopause	77		**0.036**		
No	15	Reference		Reference	
Yes	58	3.336 (0.775–14.360)	0.106	2.256 (0.282–18.033)	0.443
Unknown	4	0.000 (0.000–Inf)	0.998	0.000 (0.000–Inf)	0.999
BMI	54	0.908 (0.757–1.089)	0.298		
Chemotherapy	77		**0.029**		
No	15	Reference		Reference	
Yes	62	0.356 (0.149–0.851)	**0.020**	0.524 (0.183–1.497)	0.228
Radiotherapy	77		**0.002**		
No	42	Reference		Reference	
Yes	35	0.217 (0.073–0.641)	**0.006**	0.144 (0.044–0.464)	**0.001**
Myometrial infiltration (> = 1/2)	77		0.128		
No	25	Reference			
Yes	50	2.292 (0.765–6.870)	0.139		
Unknown	2	9.137 (0.989–84.410)	0.051		
Lymph node metastasis	77		**0.023**		
No	15	Reference		Reference	
Yes	58	6.157 (0.823–46.057)	0.077	10.666 (1.303–87.304)	**0.027**
Unknown	4	17.198 (1.535–192.721)	**0.021**	16.373 (1.302–205.940)	**0.030**
Pathological type	77		** < 0.001**		
UEC	29	Reference		Reference	
UCCC	7	3.393 (0.563–20.450)	0.182	5.367 (0.692–41.590)	0.108
USC	37	7.694 (2.206–26.830)	**0.001**	5.950 (1.613–21.951)	**0.007**
UMC	4	0.000 (0.000–Inf)	0.998	0.000 (0.000–Inf)	0.998

*UEC* uterine endometrioid carcinoma, *USC* uterine serous carcinoma, *UMC* uterine mixed carcinoma, *UCCC* uterine clear cell carcinoma, *BMI* body mass index, *OS* overall survival

The proportion of included patients receiving surgery was very high (UEC: 396/396; UCCC: 32/34; USC: 102/106; UMC: 34/34), and surgery could not be used as a prognostic factor for subsequent analysis. Therefore, patients were further divided into three different subgroups based on their postoperative adjuvant treatments: no postoperative adjuvant therapy, chemotherapy alone, or chemoradiotherapy. Compared to patients with UEC, patients with UCCC (HR = 7.414, 95%CI = 2.727–20.153,* P* < 0.001) favored poorer OS when treated with no postoperative adjuvant therapy, while patients with UCCC (HR = 104.291, 95%CI = 2.610–4167.444,* P* = 0.014) and USC (HR = 203.335, 95%CI = 8.176–5057.193,* P* = 0.001) also obtained poorer OS when treated with postoperative adjuvant chemotherapy alone (Supplementary Table S3, S4 and S5). Regarding PFS, only patients with USC (HR = 47.148, 95%CI = 5.062–439.127, *P* < 0.001) favored poorer prognosis compared to those with UEC under the treatments of postoperative adjuvant chemotherapy alone (Supplementary Table S6, S7 and S8).

## Discussion

In our study, we firstly explored the differences in clinical characteristics between three types of rare EC (UCCC, USC, and UMC) and type I EC (UEC). We found that compared to patients with UEC, patients with high-risk pathological types of EC (UCCC, USC, and UMC) were older and had a higher incidence of menopause status, which was consistent with previous research results [14, 15]. This phenomenon could be partly explained by the UEC being caused by higher estrogen exposure. Furthermore, we found that age was an independent risk factor for patients’ prognosis in the entire EC population included or some subgroup analyses. Previous studies have suggested a correlation between the occurrence of EC and high BMI [16]. However, we did not find significant differences between different pathological types in BMI. BMI was not an independent risk factor for patient prognosis in the entire EC population included, nor was it in subgroup analysis. The impact of BMI on carcinogenesis and patient prognosis in EC needs further exploration in the future.

We also explored whether different pathological types could serve as independent prognostic factors for EC. We concluded that the pathological subtypes of USC and UCCC were unfavorable prognosis factors for OS and PFS, while the UMC subtype was not. Compared to UEC, further subgroup analyses revealed that UCCC and USC were unfavorable prognosis factors for OS only in the early (stage I–II) and advanced stages (stage III–IV), respectively. On the contrary, UCCC or USC were no longer considered unfavorable prognosis factors in the early (stage I–II) and advanced (stage III–IV) stages for PFS as no significant differences were achieved in the corresponding subgroup analysis. In the future, it is necessary to collect more patients and further explore the impact of different pathological types on patients’ prognoses through more nuanced groups.

Although USC solely accounts for 10% of EC, it leads to nearly 40% of EC-related deaths [17]. Similar to previous studies, we also found that the prognosis of USC was far worse than that of UEC. In our study, the fractions of USC with stage III–IV (34.9%), myometrial infiltration (46.2%), cervix involvement (21.7%), and lymph node metastasis (29.2%) were the highest among all pathological subtypes, which could partly explain its negative effects on unfavorable prognosis. USC was an independent unfavorable prognosis factor for OS when patients were diagnosed at stage III-IV, indicating that once USC had pelvic and peritoneal metastasis, its biological behavior was closer to that of ovarian high-grade serous carcinoma, namely metastatic dissemination. Previous studies also have revealed that USC could share a similar biological behavior with advanced ovarian serous cancer, with high genomic mutation rates of HRD signaling pathway and disordered cell-cycle regulation [18–20]. All these findings may deepen the pathogenesis of USC, and contribute to finding suitable therapeutic treatments.

UCCC is another rare pathological subtype with high malignancy risk, accounting for approximately 2 to 5% of all EC cases [21, 22]. Previous studies revealed that patients with UCCC were usually diagnosed at an advanced stage, and could be susceptible to chemoresistance [12, 23]. Here, we found that among UCCC patients, 20.5% were in stage III/IV, a proportion significantly higher than that observed in patients with UEC (7.3%). Patients with UCCC had significantly poorer OS and PFS than those with UEC. Further subgroup analyses revealed that only patients with UCCC in stage I/II achieved unfavorable OS, while those with USC or UMC did not. Actually, we found that 47.8% of included patients with UCCC in stage I/II did not undergo postoperative adjuvant radiotherapy or chemotherapy, which was inconsistent with current NCCN guidelines [24]. Combined with its remarkably negative impact on the prognosis in stage I/II, we speculated that the poorer prognosis of early-stage UCCC could be due to the low proportion of postoperative adjuvant therapy, similar to some previous studies [25, 26]. Based on the above findings, our study supports the application of postoperative adjuvant treatment (chemotherapy, radiotherapy, or chemoradiotherapy) in early-stage UCCC patients. In the future, more UCCC samples should be included for further analysis.

UMC, as an extremely rare pathological type, accounting for approximately 3–8% of EC cases, has drawn attention in recent years. In 2014, the World Health Organization (WHO) defined UMC as a mixed EC composed of two or more pathological types, with at least one type II EC accounting for 10% [27]. Currently, whether the coexistence of type II EC components will affect the prognosis of patients remains elusive. A large-scale clinical study containing 934 patients compared the prognostic differences between UMC and pure USC, and no significant differences were found regarding OS and PFS [28]. The conclusions drawn from other studies with small sample sizes also varied greatly. Boruta et al. found that when the proportion of USC in UMC components was greater than 50%, patients had poorer PFS and OS [29]. Nevertheless, Nikolaos Thomakos et al. found that there was no difference in the prognosis between UMC and other type II EC, regardless of the proportions of other type II EC components in UMC [30]. In our study, we compared the prognostic differences between UMC and UEC, and we found there was no significant difference in prognosis between UMC and UEC, which may be due to the components of involved pathological types. Here, the major components of UMC were endometrioid carcinoma and other type II EC (82.35%), and the presence of endometrioid adenocarcinoma may improve the prognosis of patients to some extent. However, for those UMC patients completely composed of type II EC, it is still uncertain whether it will lead to a worse clinical prognosis due to the sample size of this study. Moreover, different molecular typing can also have a certain impact on the prognosis of patients. In the future, it is necessary to further expand the sample size and improve molecular typing for better analysis.

Although our study has concluded some novel findings, it still has its inherent limitations. Firstly, as few type II EC patients with different pathological types were included, we were unable to identify specific factors affecting patient prognosis for each pathological subtype. Secondly, the proportion of included patients receiving surgery was very high (UEC: 396/396; UCCC: 32/34; USC: 102/106; UMC: 34/34), so surgery could not be used as a prognostic factor for subsequent analysis. Thirdly, the included patients rarely received postoperative adjuvant radiotherapy alone, so radiotherapy alone could not be further analyzed in subgroup analysis. Last but not least, uterine carcinosarcoma is one of the main types of type II EC. However, we found that only limited EC patients with uterine carcinosarcoma met the inclusion criteria, and we did not include them in our study as a subgroup for subsequent analysis. We should include more eligible uterine carcinosarcomas by performing a multicenter retrospective analysis in the future.

## Conclusions

The baseline characteristics of UEC were remarkably different from those of UCCC, USC, and UMC. The prognostic significance of different pathological types on EC patients depended on clinical tumor stages and therapeutic options.

### Supplementary Information

**Additional file 1: Supplementary Table S1.** Univariate and multivariate Cox regression for PFS of stage I-II.

**Additional file 2: Supplementary Table S2.** Univariate and multivariate Cox regression for PFS of stage III-IV.

**Additional file 3: Supplementary Table S3.** Univariate and multivariate Cox regression analysis for OS in patients receiving no postoperative adjuvant therapy.

**Additional file 4: Supplementary Table S4.** Univariate and multivariate Cox regression analysis for OS in patients receiving postoperative adjuvant chemotherapy alone.

**Additional file 5: Supplementary Table S5.** Univariate and multivariate Cox regression analysis for OS in patients receiving postoperative adjuvant chemoradiotherapy.

**Additional file 6: Supplementary Table S6.** Univariate and multivariate Cox regression analysis for PFS in patients receiving no postoperative adjuvant therapy.

**Additional file 7: Supplementary Table S7.** Univariate and multivariate Cox regression analysis for PFS in patients receiving postoperative adjuvant chemotherapy.

**Additional file 8: Supplementary Table S8.** Univariate and multivariate Cox regression analysis for PFS in patients receiving postoperative adjuvant chemoradiotherapy.

## Data Availability

The data that support the findings of this study are available on request from the corresponding author for reasonable reasons. The data are not publicly available due to privacy or ethical restrictions.

## Abbreviations

EC: Endometrial carcinoma
OS: Overall survival
PFS: Progression-free survival
UEC: Uterine endometrioid carcinoma
USC: Uterine serous carcinoma
UMC: Uterine mixed carcinoma
UCCC: Uterine clear cell carcinoma
FIGO: International Federation of Gynecology and Obstetrics
