# Supplementary material for: Comparison of clinical characteristics and prognosis in endometrial carcinoma with different pathological types: a retrospective population-based study
Source: World J Surg Oncol. 2023 Nov 21;21:357. doi: 10.1186/s12957-023-03241-0 (PMC10662672; doi:10.1186/s12957-023-03241-0)
Supplement: Supplementary file 1 — Additional file 1: Supplementary Table S1. Univariate and multivariate Cox regression for PFS of stage I-II. [file 12957_2023_3241_MOESM1_ESM.docx]

**Supplementary Table 1. Univariate and multivariate Cox regression for PFS of stage I-II**

| **Characteristics** | **No.** | **Univariate analysis** | |  | **Multivariate analysis** | |
| --- | --- | --- | --- | --- | --- | --- |
|  |  | **Hazard ratio (95% CI)** | ***P*** |  | **Hazard ratio (95% CI)** | ***P*** |
| **Age** | 472 | 1.121 (1.063 - 1.183) | **< 0.001** |  | 1.208 (1.110 - 1.314) | **< 0.001** |
| **Menopause** | 472 |  | 0.073 |  |  |  |
| No | 149 | Reference |  |  | Reference |  |
| Yes | 316 | 3.922 (0.902 - 17.061) | 0.068 |  | 0.225 (0.032 - 1.593) | 0.135 |
| Unknown | 7 | 0.000 (0.000 - Inf) | 0.998 |  | 0.000 (0.000 - Inf) | 0.999 |
| **BMI** | 189 | 0.942 (0.799 - 1.109) | 0.473 |  |  |  |
| **Chemotherapy** | 472 |  | 0.266 |  |  |  |
| No | 250 | Reference |  |  |  |  |
| Yes | 222 | 1.702 (0.658 - 4.399) | 0.273 |  |  |  |
| **Radiotherapy** | 472 |  | **0.004** |  |  |  |
| No | 410 | Reference |  |  | Reference |  |
| Yes | 62 | 4.560 (1.767 - 11.766) | **0.002** |  | 2.710 (0.873 - 8.417) | 0.085 |
| **Myometrial infiltration (>=1/2)** | 472 |  | **0.002** |  |  |  |
| No | 363 | Reference |  |  | Reference |  |
| Yes | 76 | 4.752 (1.884 - 11.985) | **< 0.001** |  | 1.757 (0.439 - 7.037) | 0.426 |
| Unknown | 33 | 0.000 (0.000 - Inf) | 0.998 |  | 0.000 (0.000 - Inf) | 0.998 |
| **Cervix involvement** | 472 |  | **0.001** |  |  |  |
| No | 410 | Reference |  |  | Reference |  |
| Yes | 21 | 10.360 (3.540 - 30.315) | **< 0.001** |  | 16.569 (3.711 - 73.975) | **< 0.001** |
| Unknown | 41 | 3.014 (0.828 - 10.969) | 0.094 |  | 8.775 (1.334 - 57.713) | **0.024** |
| **Lymph node metastasis** | 472 |  | 0.475 |  |  |  |
| No | 392 | Reference |  |  |  |  |
| Unknown | 80 | 0.605 (0.139 - 2.633) | 0.503 |  |  |  |
| **Pathological type** | 472 |  | **0.006** |  |  |  |
| UEC | 362 | Reference |  |  | Reference |  |
| UCCC | 22 | 8.604 (2.270 - 32.613) | **0.002** |  | 3.272 (0.688 - 15.568) | 0.136 |
| USC | 59 | 4.737 (1.546 - 14.518) | **0.006** |  | 2.081 (0.547 - 7.924) | 0.283 |
| UMC | 29 | 3.803 (0.806 - 17.934) | 0.091 |  | 6.896 (1.078 - 44.122) | **0.041** |

UEC: Uterine Endometrioid Carcinoma; USC: Uterine Serous Carcinoma; UMC: Uterine Mixed Carcinoma; UCCC: Uterine Clear Cell Carcinoma; BMI: Body Mass Index; PFS: Progression-Free Survival.
