# Supplementary material for: Comparison of clinical characteristics and prognosis in endometrial carcinoma with different pathological types: a retrospective population-based study
Source: World J Surg Oncol. 2023 Nov 21;21:357. doi: 10.1186/s12957-023-03241-0 (PMC10662672; doi:10.1186/s12957-023-03241-0)
Supplement: Supplementary file 2 — Additional file 2: Supplementary Table S2. Univariate and multivariate Cox regression for PFS of stage III-IV. [file 12957_2023_3241_MOESM2_ESM.docx]

**Supplementary Table 2. Univariate and multivariate Cox regression for PFS of stage III-IV**

| **Characteristics** | **No.** | **Univariate analysis** | |  | **Multivariate analysis** | |
| --- | --- | --- | --- | --- | --- | --- |
|  |  | **Hazard ratio (95% CI)** | ***P*** |  | **Hazard ratio (95% CI)** | ***P*** |
| **Age** | 60 | 1.036 (0.972 - 1.105) | 0.275 |  |  |  |
| **Menopause** | 60 |  | 0.387 |  |  |  |
| No | 13 | Reference |  |  |  |  |
| Yes | 43 | 2.255 (0.277 - 18.333) | 0.447 |  |  |  |
| Unknown | 4 | 0.000 (0.000 - Inf) | 0.999 |  |  |  |
| **BMI** | 46 | 1.011 (0.839 - 1.217) | 0.911 |  |  |  |
| **Chemotherapy** | 60 |  | 0.983 |  |  |  |
| No | 8 | Reference |  |  |  |  |
| Yes | 52 | 0.978 (0.120 - 7.958) | 0.983 |  |  |  |
| **Radiotherapy** | 60 |  | 0.754 |  |  |  |
| No | 27 | Reference |  |  |  |  |
| Yes | 33 | 0.801 (0.200 - 3.203) | 0.753 |  |  |  |
| **Myometrial infiltration (>=1/2)** | 60 |  | 0.289 |  |  |  |
| No | 21 | Reference |  |  |  |  |
| Yes | 38 | 3.865 (0.475 - 31.423) | 0.206 |  |  |  |
| Unknown | 1 | 0.000 (0.000 - Inf) | 0.999 |  |  |  |
| **Lymph node metastasis** | 60 |  | 0.076 |  |  |  |
| No | 14 | Reference |  |  | Reference |  |
| Unknown | 2 | 1.002 (0.000 - Inf) | 1.000 |  | 0.815 (0.000 - Inf) | 1.000 |
| Yes | 44 | 302136183.5852 (0.000 - Inf) | 0.999 |  | 198014823.3406 (0.000 - Inf) | 0.999 |
| **Pathological type** | 60 |  | 0.069 |  |  |  |
| UEC | 27 | Reference |  |  | Reference |  |
| USC | 23 | 6.344 (0.741 - 54.337) | 0.092 |  | 4.700 (0.549 - 40.246) | 0.158 |
| UCCC | 6 | 12.230 (1.107 - 135.128) | **0.041** |  | 7.605 (0.688 - 84.013) | 0.098 |
| UMC | 4 | 0.000 (0.000 - Inf) | 0.999 |  | 0.000 (0.000 - Inf) | 0.999 |

UEC: Uterine Endometrioid Carcinoma; USC: Uterine Serous Carcinoma; UMC: Uterine Mixed Carcinoma; UCCC: Uterine Clear Cell Carcinoma; BMI: Body Mass Index; PFS: Progression-Free Survival.
