# Supplementary material for: Comparison of clinical characteristics and prognosis in endometrial carcinoma with different pathological types: a retrospective population-based study
Source: World J Surg Oncol. 2023 Nov 21;21:357. doi: 10.1186/s12957-023-03241-0 (PMC10662672; doi:10.1186/s12957-023-03241-0)
Supplement: Supplementary file 3 — Additional file 3: Supplementary Table S3. Univariate and multivariate Cox regression analysis for OS in patients receiving no postoperative adjuvant therapy. [file 12957_2023_3241_MOESM3_ESM.docx]

**Supplementary Table 3. Univariate and multivariate Cox regression analysis for OS in patients receiving no postoperative adjuvant therapy**

| **Characteristics** | **No.** | **Univariate analysis** | |  | **Multivariate analysis** | |
| --- | --- | --- | --- | --- | --- | --- |
|  |  | **Hazard ratio (95% CI)** | ***P*** |  | **Hazard ratio (95% CI)** | ***P*** |
| **Age** | 272 | 1.133 (1.084 - 1.183) | **< 0.001** |  | 1.087 (1.029 - 1.149) | **0.003** |
| **Menopause** | 272 |  | **< 0.001** |  |  |  |
| No | 98 | Reference |  |  | Reference |  |
| Yes | 169 | 408641526.9602 (0.000 - Inf) | 0.997 |  | 20418460.2921 (0.000 - Inf) | 0.997 |
| Unknown | 5 | 1.015 (0.000 - Inf) | 1.000 |  | 0.977 (0.000 - Inf) | 1.000 |
| **Stage** | 272 |  | **< 0.001** |  |  |  |
| I | 249 | Reference |  |  | Reference |  |
| II | 4 | 4.856 (0.627 - 37.602) | 0.130 |  | 2.030 (0.267 - 15.446) | 0.494 |
| III | 10 | 15.695 (5.859 - 42.049) | **< 0.001** |  | 0.538 (0.208 - 1.387) | 0.199 |
| IV | 4 | 19.669 (4.275 - 90.492) | **< 0.001** |  | 8.948 (1.811 - 44.217) | **0.007** |
| Unknown | 5 | 9.798 (2.155 - 44.553) | **0.003** |  | 0.818 (0.184 - 3.635) | 0.792 |
| **Myometrial infiltration (>=1/2)** | 272 |  | **< 0.001** |  |  |  |
| No | 215 | Reference |  |  | Reference |  |
| Yes | 23 | 16.538 (6.735 - 40.608) | **< 0.001** |  | 11.776 (5.072 - 27.337) | **< 0.001** |
| Unknown | 34 | 2.719 (0.719 - 10.279) | 0.140 |  | 1.520 (0.439 - 5.265) | 0.509 |
| **Cervix involvement** | 272 |  | **0.001** |  |  |  |
| No | 231 | Reference |  |  | Reference |  |
| Yes | 9 | 9.690 (3.451 - 27.206) | **< 0.001** |  | 0.242 (0.087 - 0.676) | **0.007** |
| Unknown | 32 | 2.332 (0.764 - 7.119) | 0.137 |  | 1.581 (0.521 - 4.794) | 0.419 |
| **Lymph node metastasis** | 272 |  | **< 0.001** |  |  |  |
| No | 193 | Reference |  |  | Reference |  |
| Yes | 8 | 27.196 (10.016 - 73.839) | **< 0.001** |  | 15.071 (5.919 - 38.369) | **< 0.001** |
| Unknown | 71 | 2.155 (0.802 - 5.790) | 0.128 |  | 2.391 (0.959 - 5.964) | 0.062 |
| **Pathological type** | 272 |  | **< 0.001** |  |  |  |
| UEC | 228 | Reference |  |  | Reference |  |
| UCCC | 14 | 12.168 (3.969 - 37.301) | **< 0.001** |  | 7.414 (2.727 - 20.153) | **< 0.001** |
| USC | 24 | 14.333 (5.492 - 37.406) | **< 0.001** |  | 1.254 (0.520 - 3.021) | 0.615 |
| UMC | 6 | 6.164 (0.767 - 49.535) | 0.087 |  | 0.404 (0.053 - 3.103) | 0.384 |

UEC: Uterine Endometrioid Carcinoma; USC: Uterine Serous Carcinoma; UMC: Uterine Mixed Carcinoma; UCCC: Uterine Clear Cell Carcinoma; BMI: Body Mass Index; OS: Overall Survival.
