# Supplementary material for: Comparison of clinical characteristics and prognosis in endometrial carcinoma with different pathological types: a retrospective population-based study
Source: World J Surg Oncol. 2023 Nov 21;21:357. doi: 10.1186/s12957-023-03241-0 (PMC10662672; doi:10.1186/s12957-023-03241-0)
Supplement: Supplementary file 4 — Additional file 4: Supplementary Table S4. Univariate and multivariate Cox regression analysis for OS in patients receiving postoperative adjuvant chemotherapy alone. [file 12957_2023_3241_MOESM4_ESM.docx]

**Supplementary Table 4. Univariate and multivariate Cox regression analysis for OS in patients receiving postoperative adjuvant chemotherapy alone**

| **Characteristics** | **No.** | **Univariate analysis** | |  | **Multivariate analysis** | |
| --- | --- | --- | --- | --- | --- | --- |
|  |  | **Hazard ratio (95% CI)** | ***P*** |  | **Hazard ratio (95% CI)** | ***P*** |
| **Age** | 198 | 1.088 (1.028 - 1.151) | **0.004** |  | 1.002 (0.925 - 1.085) | 0.966 |
| **Menopause** | 198 |  | 0.205 |  |  |  |
| No | 44 | Reference |  |  |  |  |
| Yes | 151 | 4.093 (0.535 - 31.318) | 0.175 |  |  |  |
| Unknown | 3 | 0.000 (0.000 - Inf) | 0.998 |  |  |  |
| **BMI** | 159 | 1.048 (0.909 - 1.207) | 0.519 |  |  |  |
| **Stage** | 198 |  | **< 0.001** |  |  |  |
| I | 163 | Reference |  |  | Reference |  |
| II | 6 | 0.000 (0.000 - Inf) | 0.999 |  | 0.000 (0.000 - Inf) | 0.999 |
| III | 21 | 18.047 (4.499 - 72.395) | **< 0.001** |  | 1.161 (0.063 - 21.431) | 0.920 |
| IV | 7 | 63.442 (13.565 - 296.712) | **< 0.001** |  | 2.769 (0.127 - 60.276) | 0.517 |
| Unknown | 1 |  |  |  |  |  |
| **Myometrial infiltration (>=1/2)** | 198 |  | **0.010** |  |  |  |
| No | 127 | Reference |  |  | Reference |  |
| Yes | 64 | 4.061 (1.223 - 13.487) | **0.022** |  | 1.894 (0.299 - 12.009) | 0.498 |
| Unknown | 7 | 11.998 (2.196 - 65.569) | **0.004** |  | 0.286 (0.011 - 7.373) | 0.451 |
| **Cervix involvement** | 198 |  | **0.016** |  |  |  |
| No | 163 | Reference |  |  | Reference |  |
| Yes | 20 | 5.015 (1.467 - 17.148) | **0.010** |  | 1.746 (0.356 - 8.557) | 0.492 |
| Unknown | 15 | 5.057 (1.307 - 19.564) | **0.019** |  | 20.690 (0.809 - 529.125) | 0.067 |
| **Lymph node metastasis** | 198 |  | **< 0.001** |  |  |  |
| No | 159 | Reference |  |  | Reference |  |
| Yes | 19 | 87.684 (10.916 - 704.364) | **< 0.001** |  | 15.884 (0.432 - 584.151) | 0.133 |
| Unknown | 20 | 45.712 (5.339 - 391.361) | **< 0.001** |  | 86.519 (4.487 - 1668.449) | **0.003** |
| **Pathological type** | 198 |  | **< 0.001** |  |  |  |
| UEC | 132 | Reference |  |  | Reference |  |
| UCCC | 9 | 23.978 (3.315 - 173.444) | **0.002** |  | 104.291 (2.610 - 4167.444) | **0.014** |
| USC | 39 | 23.263 (5.022 - 107.767) | **< 0.001** |  | 203.335 (8.176 - 5057.193) | **0.001** |
| UMC | 18 | 0.000 (0.000 - Inf) | 0.998 |  | 0.000 (0.000 - Inf) | 0.999 |

UEC: Uterine Endometrioid Carcinoma; USC: Uterine Serous Carcinoma; UMC: Uterine Mixed Carcinoma; UCCC: Uterine Clear Cell Carcinoma; BMI: Body Mass Index; OS: Overall Survival.
