# Supplementary material for: Comparison of clinical characteristics and prognosis in endometrial carcinoma with different pathological types: a retrospective population-based study
Source: World J Surg Oncol. 2023 Nov 21;21:357. doi: 10.1186/s12957-023-03241-0 (PMC10662672; doi:10.1186/s12957-023-03241-0)
Supplement: Supplementary file 5 — Additional file 5: Supplementary Table S5. Univariate and multivariate Cox regression analysis for OS in patients receiving postoperative adjuvant chemoradiotherapy. [file 12957_2023_3241_MOESM5_ESM.docx]

**Supplementary Table 5. Univariate and multivariate Cox regression analysis for OS in patients receiving postoperative adjuvant chemoradiotherapy**

| **Characteristics** | **No.** | **Univariate analysis** | |  | **Multivariate analysis** | |
| --- | --- | --- | --- | --- | --- | --- |
|  |  | **Hazard ratio (95% CI)** | ***P*** |  | **Hazard ratio (95% CI)** | ***P*** |
| **Age** | 95 | 1.055 (0.979 - 1.136) | 0.161 |  |  |  |
| **Menopause** | 95 |  | 0.221 |  |  |  |
| No | 22 | Reference |  |  |  |  |
| Yes | 69 | 3.266 (0.413 - 25.806) | 0.262 |  |  |  |
| Unknown | 4 | 0.000 (0.000 - Inf) | 0.998 |  |  |  |
| **BMI** | 77 | 0.883 (0.719 - 1.085) | 0.237 |  |  |  |
| **Stage** | 95 |  | 0.102 |  |  |  |
| I | 54 | Reference |  |  |  |  |
| III | 31 | 0.739 (0.143 - 3.812) | 0.718 |  |  |  |
| IV | 3 | 19.441 (2.989 - 126.453) | **0.002** |  |  |  |
| II | 6 | 1.556 (0.181 - 13.340) | 0.687 |  |  |  |
| Unknown | 1 | 0.000 (0.000 - Inf) | 0.999 |  |  |  |
| **Myometrial infiltration (>=1/2)** | 95 |  | 0.090 |  |  |  |
| No | 48 | Reference |  |  | Reference |  |
| Yes | 45 | 4.381 (0.928 - 20.676) | 0.062 |  | 4.381 (0.928 - 20.676) | 0.062 |
| Unknown | 2 | 0.000 (0.000 - Inf) | 0.998 |  | 0.000 (0.000 - Inf) | 0.998 |
| **Cervix involvement** | 95 |  | 0.605 |  |  |  |
| No | 70 | Reference |  |  |  |  |
| Yes | 19 | 2.031 (0.507 - 8.137) | 0.317 |  |  |  |
| Unknown | 6 | 1.685 (0.202 - 14.049) | 0.630 |  |  |  |
| **Lymph node metastasis** | 95 |  | 0.542 |  |  |  |
| No | 61 | Reference |  |  |  |  |
| Yes | 30 | 1.564 (0.439 - 5.569) | 0.490 |  |  |  |
| Unknown | 4 | 0.000 (0.000 - Inf) | 0.998 |  |  |  |
| **Pathological type** | 95 |  | 0.150 |  |  |  |
| UEC | 33 | Reference |  |  |  |  |
| UCCC | 11 | 4.211 (0.588 - 30.156) | 0.152 |  |  |  |
| USC | 42 | 3.279 (0.658 - 16.347) | 0.147 |  |  |  |
| UMC | 9 | 0.000 (0.000 - Inf) | 0.998 |  |  |  |

UEC: Uterine Endometrioid Carcinoma; USC: Uterine Serous Carcinoma; UMC: Uterine Mixed Carcinoma; UCCC: Uterine Clear Cell Carcinoma; BMI: Body Mass Index; OS: Overall Survival.
