# Supplementary material for: Comparison of clinical characteristics and prognosis in endometrial carcinoma with different pathological types: a retrospective population-based study
Source: World J Surg Oncol. 2023 Nov 21;21:357. doi: 10.1186/s12957-023-03241-0 (PMC10662672; doi:10.1186/s12957-023-03241-0)
Supplement: Supplementary file 6 — Additional file 6: Supplementary Table S6. Univariate and multivariate Cox regression analysis for PFS in patients receiving no postoperative adjuvant therapy. [file 12957_2023_3241_MOESM6_ESM.docx]

**Supplementary Table 6. Univariate and multivariate Cox regression analysis for PFS in patients receiving no postoperative adjuvant therapy**

| **Characteristics** | **No.** | **Univariate analysis** | |  | **Multivariate analysis** | |
| --- | --- | --- | --- | --- | --- | --- |
|  |  | **Hazard ratio (95% CI)** | ***P*** |  | **Hazard ratio (95% CI)** | ***P*** |
| **Age** | 256 | 1.116 (1.039 - 1.199) | **0.003** |  | 1.136 (1.038 - 1.243) | **0.005** |
| **Menopause** | 256 |  | 0.200 |  |  |  |
| No | 98 | Reference |  |  |  |  |
| Yes | 153 | 4.624 (0.569 - 37.596) | 0.152 |  |  |  |
| Unknown | 5 | 0.000 (0.000 - Inf) | 0.999 |  |  |  |
| **Stage** | 256 |  | 0.561 |  |  |  |
| I | 243 | Reference |  |  |  |  |
| II | 3 | 0.000 (0.000 - Inf) | 0.999 |  |  |  |
| III | 5 | 9.207 (1.126 - 75.287) | **0.038** |  |  |  |
| IV | 2 | 0.000 (0.000 - Inf) | 0.999 |  |  |  |
| Unknown | 3 | 0.000 (0.000 - Inf) | 0.999 |  |  |  |
| **Myometrial infiltration (>=1/2)** | 256 |  | **0.013** |  |  |  |
| No | 210 | Reference |  |  | Reference |  |
| Yes | 15 | 9.306 (2.219 - 39.026) | **0.002** |  | 0.000 (0.000 - 0.000) | **< 0.001** |
| Unknown | 31 | 0.000 (0.000 - Inf) | 0.999 |  | 0.000 (0.000 - Inf) | 0.995 |
| **Cervix involvement** | 256 |  | **0.020** |  |  |  |
| No | 221 | Reference |  |  | Reference |  |
| Yes | 6 | 21.599 (4.171 - 111.848) | **< 0.001** |  | 17752195264450960.0000 (2110746054174650.0000 - 149302866673093088.0000) | **< 0.001** |
| Unknown | 29 | 1.731 (0.200 - 14.979) | 0.618 |  | 16186662225076890.0000 (1889109964190685.0000 - 138693902925327184.0000) | **< 0.001** |
| **Lymph node metastasis** | 256 |  | 0.088 |  |  |  |
| No | 189 | Reference |  |  | Reference |  |
| Yes | 2 | 21.915 (2.588 - 185.611) | **0.005** |  | 741302919.0299 (0.000 - Inf) | 0.996 |
| Unknown | 65 | 0.501 (0.060 - 4.160) | 0.522 |  | 0.486 (0.058 - 4.065) | 0.506 |
| **Pathological type** | 256 |  | 0.247 |  |  |  |
| UEC | 224 | Reference |  |  |  |  |
| UCCC | 11 | 3.993 (0.481 - 33.181) | 0.200 |  |  |  |
| USC | 16 | 0.000 (0.000 - Inf) | 0.998 |  |  |  |
| UMC | 5 | 8.235 (0.989 - 68.575) | 0.051 |  |  |  |

UEC: Uterine Endometrioid Carcinoma; USC: Uterine Serous Carcinoma; UMC: Uterine Mixed Carcinoma; UCCC: Uterine Clear Cell Carcinoma; BMI: Body Mass Index; PFS: Progression-Free Survival.
