# Supplementary material for: Comparison of clinical characteristics and prognosis in endometrial carcinoma with different pathological types: a retrospective population-based study
Source: World J Surg Oncol. 2023 Nov 21;21:357. doi: 10.1186/s12957-023-03241-0 (PMC10662672; doi:10.1186/s12957-023-03241-0)
Supplement: Supplementary file 7 — Additional file 7: Supplementary Table S7. Univariate and multivariate Cox regression analysis for PFS in patients receiving postoperative adjuvant chemotherapy. [file 12957_2023_3241_MOESM7_ESM.docx]

**Supplementary Table 7. Univariate and multivariate Cox regression analysis for PFS in patients receiving postoperative adjuvant chemotherapy**

| **Characteristics** | **No.** | **Univariate analysis** | |  | **Multivariate analysis** | |
| --- | --- | --- | --- | --- | --- | --- |
|  |  | **Hazard ratio (95% CI)** | ***P*** |  | **Hazard ratio (95% CI)** | ***P*** |
| **Age** | 184 | 1.108 (1.024 - 1.198) | **0.011** |  | 1.085 (0.967 - 1.216) | 0.165 |
| **Menopause** | 184 |  | 0.122 |  |  |  |
| No | 42 | Reference |  |  |  |  |
| Yes | 140 | 292106587.3964 (0.000 - Inf) | 0.999 |  |  |  |
| Unknown | 2 | 0.995 (0.000 - Inf) | 1.000 |  |  |  |
| **BMI** | 147 | 0.857 (0.685 - 1.074) | 0.180 |  |  |  |
| **Stage** | 184 |  | **0.035** |  |  |  |
| I | 158 | Reference |  |  | Reference |  |
| II | 6 | 8.908 (0.926 - 85.686) | 0.058 |  | 0.921 (0.100 - 8.488) | 0.942 |
| III | 17 | 10.749 (2.162 - 53.425) | **0.004** |  | 0.000 (0.000 - 0.000) | **< 0.001** |
| IV | 3 | 0.000 (0.000 - Inf) | 0.999 |  | 0.000 (0.000 - Inf) | 1.000 |
| **Myometrial infiltration (>=1/2)** | 184 |  | 0.085 |  |  |  |
| No | 122 | Reference |  |  | Reference |  |
| Yes | 57 | 5.296 (1.027 - 27.306) | **0.046** |  | 4.055 (0.699 - 23.524) | 0.119 |
| Unknown | 5 | 0.000 (0.000 - Inf) | 0.999 |  | 0.000 (0.000 - Inf) | 0.999 |
| **Cervix involvement** | 184 |  | 0.221 |  |  |  |
| No | 155 | Reference |  |  |  |  |
| Yes | 17 | 4.810 (0.880 - 26.302) | 0.070 |  |  |  |
| Unknown | 12 | 2.911 (0.325 - 26.096) | 0.340 |  |  |  |
| **Lymph node metastasis** | 184 |  | **0.010** |  |  |  |
| No | 156 | Reference |  |  | Reference |  |
| Yes | 13 | 16.172 (3.217 - 81.290) | **< 0.001** |  | 158010021.2646 (22577777.0968 - 1105829272.4282) | **< 0.001** |
| Unknown | 15 | 3.359 (0.349 - 32.303) | 0.294 |  | 14.481 (1.562 - 134.211) | **0.019** |
| **Pathological type** | 184 |  | **< 0.001** |  |  |  |
| UEC | 129 | Reference |  |  | Reference |  |
| UCCC | 7 | 0.000 (0.000 - Inf) | 0.999 |  | 0.000 (0.000 - Inf) | 0.998 |
| USC | 30 | 37.938 (4.493 - 320.342) | **< 0.001** |  | 47.148 (5.062 - 439.127) | **< 0.001** |
| UMC | 18 | 0.000 (0.000 - Inf) | 0.999 |  | 0.000 (0.000 - Inf) | 0.999 |

UEC: Uterine Endometrioid Carcinoma; USC: Uterine Serous Carcinoma; UMC: Uterine Mixed Carcinoma; UCCC: Uterine Clear Cell Carcinoma; BMI: Body Mass Index; PFS: Progression-Free Survival.
