# Supplementary material for: Comparison of clinical characteristics and prognosis in endometrial carcinoma with different pathological types: a retrospective population-based study
Source: World J Surg Oncol. 2023 Nov 21;21:357. doi: 10.1186/s12957-023-03241-0 (PMC10662672; doi:10.1186/s12957-023-03241-0)
Supplement: Supplementary file 8 — Additional file 8: Supplementary Table S8. Univariate and multivariate Cox regression analysis for PFS in patients receiving postoperative adjuvant chemoradiotherapy. [file 12957_2023_3241_MOESM8_ESM.docx]

**Supplementary Table 8. Univariate and multivariate Cox regression analysis for PFS in patients receiving postoperative adjuvant chemoradiotherapy**

| **Characteristics** | **No.** | **Univariate analysis** | |  | **Multivariate analysis** | |
| --- | --- | --- | --- | --- | --- | --- |
|  |  | **Hazard ratio (95% CI)** | ***P*** |  | **Hazard ratio (95% CI)** | ***P*** |
| **Age** | 91 | 1.077 (1.003 - 1.156) | **0.042** |  | 1.032 (0.968 - 1.101) | 0.331 |
| **Menopause** | 91 |  | 0.494 |  |  |  |
| No | 21 | Reference |  |  |  |  |
| Yes | 66 | 1.500 (0.323 - 6.958) | 0.605 |  |  |  |
| Unknown | 4 | 0.000 (0.000 - Inf) | 0.998 |  |  |  |
| **BMI** | 73 | 1.042 (0.892 - 1.219) | 0.602 |  |  |  |
| **Stage** | 91 |  | 0.766 |  |  |  |
| I | 52 | Reference |  |  |  |  |
| II | 6 | 1.416 (0.170 - 11.767) | 0.748 |  |  |  |
| III | 30 | 0.869 (0.217 - 3.475) | 0.843 |  |  |  |
| IV | 2 | 4.558 (0.543 - 38.243) | 0.162 |  |  |  |
| Unknown | 1 | 0.000 (0.000 - Inf) | 0.998 |  |  |  |
| **Myometrial infiltration (>=1/2)** | 91 |  | 0.145 |  |  |  |
| No | 48 | Reference |  |  |  |  |
| Yes | 41 | 3.195 (0.847 - 12.047) | 0.086 |  |  |  |
| Unknown | 2 | 0.000 (0.000 - Inf) | 0.998 |  |  |  |
| **Cervix involvement** | 91 |  | **0.020** |  |  |  |
| No | 67 | Reference |  |  | Reference |  |
| Yes | 18 | 6.137 (1.730 - 21.761) | **0.005** |  | 4.750 (1.279 - 17.637) | **0.020** |
| Unknown | 6 | 2.575 (0.287 - 23.073) | 0.398 |  | 4.269 (0.389 - 46.905) | 0.235 |
| **Lymph node metastasis** | 91 |  | 0.577 |  |  |  |
| No | 59 | Reference |  |  |  |  |
| Yes | 28 | 1.234 (0.361 - 4.218) | 0.738 |  |  |  |
| Unknown | 4 | 0.000 (0.000 - Inf) | 0.998 |  |  |  |
| **Pathological type** | 91 |  | 0.098 |  |  |  |
| UEC | 33 | Reference |  |  | Reference |  |
| USC | 38 | 1.860 (0.339 - 10.197) | 0.475 |  | 1.864 (0.292 - 11.905) | 0.510 |
| UCCC | 11 | 8.057 (1.468 - 44.211) | **0.016** |  | 5.427 (0.728 - 40.469) | 0.099 |
| UMC | 9 | 2.060 (0.187 - 22.734) | 0.555 |  | 2.988 (0.238 - 37.580) | 0.397 |

UEC: Uterine Endometrioid Carcinoma; USC: Uterine Serous Carcinoma; UMC: Uterine Mixed Carcinoma; UCCC: Uterine Clear Cell Carcinoma; BMI: Body Mass Index; PFS: Progression-Free Survival.
